# Dronedarone treatment following cardioversion in patients with atrial fibrillation/flutter: A post hoc analysis of the EURIDIS and ADONIS trials

**DOI:** 10.1111/jce.14405

**Published:** 2020-03-05

**Authors:** Munveer Thind, Harry J. Crijns, Gerald V. Naccarelli, James A. Reiffel, Valérie Corp dit Genti, Mattias Wieloch, Andrew Koren, Peter R. Kowey

**Affiliations:** ^1^ Division of Cardiovascular Medicine Lankenau Heart Institute Wynnewood Pennsylvania; ^2^ Department of Cardiology Maastricht University Medical Center and CARIM Maastricht Netherlands; ^3^ Department of Medicine, Division of Cardiology Penn State University College of Medicine Hershey Pennsylvania; ^4^ Department of Medicine, Division of Cardiology, Vagelos College of Physicians and Surgeons Columbia University New York New York; ^5^ Sanofi‐Aventis Paris France; ^6^ Department of Clinical Sciences Malmö Lund University Lund Sweden; ^7^ Sanofi US Inc Bridgewater New Jersey

**Keywords:** antiarrhythmic drug, atrial fibrillation, atrial flutter, cardioversion, dronedarone

## Abstract

**Introduction:**

The phase 3 EURIDIS and ADONIS studies evaluated dronedarone for atrial fibrillation (AF)/atrial flutter (AFL) recurrence in patients with nonpermanent AF. Here we assessed whether patient characteristics and/or treatment outcomes in these studies differed based on the need for cardioversion before randomization.

**Methods:**

Time to adjudicated first AF/AFL recurrence, symptomatic recurrence, cardiovascular hospitalization/death, and AF hospitalization, and safety were assessed by cardioversion status.

**Results:**

Of 1237 patients randomized (2:1 dronedarone:placebo), 364 required baseline cardioversion (dronedarone 243, placebo 121). Patients requiring cardioversion had a greater prevalence of cardiovascular comorbidities and shorter times to first AF/AFL recurrence compared with those not requiring cardioversion. Dronedarone was associated with longer median time to first AF/AFL recurrence vs placebo regardless of cardioversion status (cardioversion: 50 vs 15 days, hazard ratio [HR] 0.76; 95% confidence interval [CI], 0.59‐0.97; *P* = .02; non‐cardioversion: 150 vs 77 days, HR 0.76; 95% CI, 0.64‐0.90; *P* < .01). Dronedarone was similarly associated with prolonged median time to symptomatic recurrence vs placebo in the cardioversion (347 vs 87 days, HR 0.65; 95% CI, 0.49‐0.87) and non‐cardioversion (288 vs 120 days, HR 0.74; 95% CI, 0.62‐0.90) populations. Risk of cardiovascular hospitalization/death and first AF hospitalization was lower with dronedarone vs placebo regardless of cardioversion status, but differences were not statistically significant. The safety of dronedarone was similar in both groups.

**Conclusion:**

Patients requiring baseline cardioversion represent a distinct population, having more underlying cardiovascular disease and experiencing a shorter time to AF/AFL recurrences. Dronedarone was associated with improved efficacy vs placebo regardless of cardioversion status.

## 1 INTRODUCTION

In clinical practice, antiarrhythmic drugs (AADs) are typically not recommended after the first diagnosis of atrial fibrillation (AF). Rather, the decision to use AADs is made based on AF tolerance (symptoms, hemodynamics), the likelihood of AF recurrence, and/or the need for cardioversion to manage recurrences. Clinical trials evaluating the safety and efficacy of AADs in paroxysmal/persistent AF often include a mix of patients who are in sinus rhythm, require cardioversion before treatment or are assessed for pharmacologic cardioversion.^1^, ^2^, ^3^, ^4^, ^5^, ^6^ Baseline characteristics, comorbidities, and disease burden of patients requiring cardioversion for AF may differ from those who do not require cardioversion, as may responses to AAD therapy. Thus, cardioversion status in patients being considered for treatment with AADs is an important consideration in clinical practice; however, little information is available on this subject.

Dronedarone is an AAD indicated to reduce risk of hospitalization for AF in patients in sinus rhythm with a history of paroxysmal/persistent AF.^7^ In the European Trial in AF or Flutter Patients Receiving Dronedarone for the Maintenance of Sinus Rhythm (EURIDIS; NCT00259428) and the American‐Australian‐African Trial with Dronedarone in AF Patients for the Maintenance of Sinus Rhythm (ADONIS; NCT00259376), dronedarone significantly increased the time to first documented AF/atrial flutter (AFL) recurrence and reduced ventricular rate during recurrence compared with placebo.^8^ In these trials, patients in AF/AFL were included if they reverted to sinus rhythm or had successful cardioversion within 5 days before randomization.^8^ In this pooled analysis of EURIDIS and ADONIS, we assessed whether baseline characteristics and treatment outcomes differed by baseline cardioversion status.

## 2 METHODS

### 2.1 Overview of the EURIDIS and ADONIS studies

EURIDIS and ADONIS were concurrent and identically designed double‐blind, randomized, multicenter, phase 3 studies conducted to evaluate the safety and efficacy of dronedarone in maintaining sinus rhythm in individuals with nonpermanent AF/AFL; the eligibility criteria and design of these trials have been described previously.^8^ The studies enrolled patients who experienced at least one episode of AF/AFL observed on electrocardiogram (ECG) in the preceding 3 months.^8^ All patients were required to have been in sinus rhythm for ≥1 hour to be eligible for randomization.^8^ Eligible patients not in sinus rhythm during the 7‐day screening period were permitted to participate in the studies if they underwent successful cardioversion (electrical or with ibutilide) within 5 days before randomization and remained in sinus rhythm for ≥1 hour.^8^ Concomitant treatment with Vaughan‐Williams class I or III AADs was not allowed.

Patients were randomized in a 2:1 ratio to oral dronedarone 400 mg twice daily or placebo for 12 months.^8^ A dynamic allocation was performed to balance treatment groups with regard to prognostic factors, study center, need for baseline cardioversion, and chronic treatment with amiodarone before randomization. The primary endpoint of the EURIDIS/ADONIS studies was time to first documented recurrence of AF/AFL within 12 months. Secondary endpoints included symptomatic AF/AFL recurrence and mean ventricular rate during the first recurrence. AF/AFL recurrence was defined as an episode lasting for ≥10 minutes and confirmed by two consecutive recordings taken 10 minutes apart on 12‐lead ECG or trans‐telephonic ECG monitoring (TTEM). AF/AFL recurrence was evaluated centrally by scheduled TTEM (on days 2, 3, and 5; months 3, 5, 7, and 10; and at symptom recurrence), as well as 12‐lead ECG, obtained during study visits on days 7, 14, and 21, and months 2, 4, 6, 9, and 12.^8^

Safety was assessed via an adverse event (AE) reporting, vital signs, ECGs, and laboratory evaluations. AEs were categorized according to the Medical Dictionary for Regulatory Activities (versions 6.0 and 6.1) dictionary terms, consistent with regulatory agency guidance. Treatment‐emergent AEs (TEAEs) were defined as AEs that occurred or worsened during study treatment or within 10 days following the last drug intake.

### 2.2 Pooled analysis

We performed a post hoc analysis of the EURIDIS/ADONIS trials by baseline cardioversion status. Baseline demographic and cardiovascular disease‐related characteristics (including scores on the congestive heart failure, hypertension, age ≥75 years [doubled], diabetes mellitus, prior stroke or transient ischemic attack [doubled], vascular disease, age 65 to 74 years, sex category: female [CHA_2_DS_2_‐VASc]^9^ index, which were not assessed in the primary analysis of the EURIDIS/ADONIS studies) are summarized. Sinus rates at baseline were recorded using 12‐lead ECG. The EURIDIS/ADONIS primary endpoint of time to first documented recurrence of AF/AFL within 12 months was evaluated retrospectively in the cardioversion and non‐cardioversion groups. The following outcomes were also analyzed: time to symptomatic first AF/AFL recurrence, symptoms at first recurrence, ventricular rates during adjudicated and symptomatic first recurrence, time to first cardiovascular hospitalization or death, and time to first AF hospitalization.

### 2.3 Statistical analysis

The analysis was performed in all randomized patients who received at least one dose of study drug. Baseline data in the treatment arms of each group are descriptive.

The nonparametric Kaplan–Meier method was used to estimate cumulative incidence functions for time to events in the cardioversion and non‐cardioversion groups. Within each cardioversion group, time‐to‐event endpoints were compared between dronedarone and placebo treatment groups using a 2‐sided log‐rank asymptotic test. Hazard ratios (HRs) and 95% confidence intervals (CIs) were estimated using a Cox model with study treatment as the only factor. Since the distribution of demographic characteristics was balanced in the groups, it was not considered necessary to adjust for baseline covariates in the model. Data were analyzed with SAS version 9.4 (Cary, NC).

## 3 RESULTS

### 3.1 Baseline demographics and patient characteristics

In the EURIDIS and ADONIS studies, a total of 1237 patients were randomized to and received treatment with dronedarone (n = 828) or placebo (n = 409). Of these, 364 (29.4%) patients required cardioversion for study entry: 243 in the dronedarone arm (29.3%) and 121 in the placebo arm (29.6%).

Patients requiring cardioversion tended to have a higher prevalence of structural heart disease (driven by valvular and rheumatic heart disease), congestive heart failure, and greater left atrial diameter compared with patients not requiring cardioversion (Table 1). A CHA_2_DS_2_‐VASc score of ≥2 was observed in 63.2% of patients who underwent cardioversion vs 56.5% of those who did not. Sinus rates at baseline were similar regardless of treatment group or cardioversion status. Mean values ranged between 63 beats per minute (bpm) and 65 bpm with SD values between 10 and 11 bpm; median values ranged between 61 bpm and 63 bpm with minimum and maximum values between 42 bpm and 113 bpm, respectively. Mean (SD) ventricular rates at last AF/AFL before randomization were 93 bpm (27) for dronedarone and 96 bpm (27) for placebo in the cardioversion group and 108 bpm (30) for dronedarone and 109 bpm (33) for placebo in the non‐cardioversion group. Corresponding median (range) values were 89 bpm (47‐174) for dronedarone and 89 bpm (50‐193) for placebo in the cardioversion group and 105 bpm (45‐215) for dronedarone and 104 bpm (45‐199) for placebo in the non‐cardioversion group.

**Table 1:** Baseline characteristics

	Cardioversion			Non‐cardioversion		
Parameter	Dronedarone (n = 243)	Placebo (n = 121)	Total (n = 364)	Dronedarone (n = 585)	Placebo (n = 288)	Total (n = 873)
Study, n						
EURIDIS	153	75	228	258	126	384
ADONIS	90	46	136	327	162	489
Patient characteristics						
Male, n (%)	177 (72.8)	87 (71.9)	264 (72.5)	401 (68.5)	193 (67.0)	594 (68.0)
Age, mean (SD), y	64.6 (9.5)	62.2 (11.1)	63.8 (10.1)	63.0 (11.2)	62.2 (11.1)	62.7 (11.2)
Race, White, n (%)	238 (97.9)	117 (96.7)	355 (97.5)	562 (96.1)	283 (98.3)	845 (96.8)
BMI, mean (SD), kg/m^2,^ ^a^	28.8 (5.2)	29.0 (5.1)	28.9 (5.1)	28.8 (5.3)	28.8 (5.0)	28.8 (5.2)
CHA_2_DS_2_‐VASc score, n (%)^b^						
0‐1	84 (34.6)	50 (41.3)	134 (36.8)	246 (42.1)	134 (46.5)	380 (43.5)
2‐3	121 (49.8)	50 (41.3)	171 (47.0)	249 (42.6)	112 (38.9)	361 (41.4)
>3	38 (15.6)	21 (17.4)	59 (16.2)	90 (15.4)	42 (14.6)	132 (15.1)
Comorbidities						
Cardiovascular history, n (%)						
Structural heart disease^a^	110 (45.6)	53 (45.7)	163 (45.7)	238 (41.1)	106 (37.2)	344 (39.8)
Hypertension	147 (60.5)	64 (52.9)	211 (58.0)	350 (59.8)	141 (49.0)	491 (56.2)
Coronary artery disease^c^	57 (23.5)	24 (19.8)	81 (22.3)	138 (23.6)	51 (17.7)	189 (21.6)
Cardiac valvular disease	58 (23.9)	20 (16.5)	78 (21.4)	78 (13.3)	41 (14.2)	119 (13.6)
Dilated cardiomyopathy	16 (6.6)	13 (10.7)	29 (8.0)	34 (5.8)	17 (5.9)	51 (5.8)
Implanted pacemaker	21 (8.6)	7 (5.8)	28 (7.7)	43 (7.4)	13 (4.5)	56 (6.4)
Implanted cardioverter‐defibrillator	3 (1.2)	2 (1.7)	5 (1.4)	3 (0.5)	3 (1.0)	6 (0.7)
Rheumatic heart disease	15 (6.2)	7 (5.8)	22 (6.0)	10 (1.7)	7 (2.4)	17 (1.9)
LV ejection fraction, mean (SD), %	56.7 (11.0)	55.8 (12.8)	56.4 (11.6)	59.6 (10.6)	59.6 (9.9)	59.6 (10.4)
<35%, n (%)^a^	9 (3.9)	8 (7.0)	17 (4.9)	14 (2.5)	8 (3.0)	22 (2.6)
Left atrium diameter, mean (SD), mm^a^	44.8 (6.9)	45.2 (7.2)	44.9 (7.0)	41.7 (6.8)	41.2 (6.3)	41.6 (6.7)
Congestive heart failure, n (%)^d^						
NYHA class I	12 (4.9)	10 (8.3)	22 (6.0)	35 (6.0)	16 (5.6)	51 (5.8)
NYHA class II	39 (16.0)	21 (17.4)	60 (16.5)	57 (9.7)	26 (9.0)	83 (9.5)

*Notes*: CHA_2_DS_2_‐VASc, congestive heart failure, hypertension, age ≥75 y (doubled), diabetes mellitus, prior stroke or transient ischemic attack (doubled), vascular disease, age 65‐74 y, sex category: female.

Abbreviations: BMI, body mass index; LV, left ventricular; NYHA, New York Heart Association; SD, standard deviation.

^a^ Data were missing for <8% of patients; percentages are calculated as a proportion of all patients with available data.

^b^ Derived a posteriori (not included in the primary analysis of the EURIDIS and ADONIS studies).

^c^ The diagnosis of coronary artery disease was made on the basis of the clinical history and the results of investigational tests.

^d^ The diagnosis of congestive heart failure (NYHA class I and II) was made on clinical grounds. Patients who were classified as having NYHA class I congestive heart failure had received a diagnosis of the disease but had no symptoms.

Prior and concomitant use of oral anticoagulant and rate‐controlling medications at baseline differed by cardioversion status (Table 2). The use of anticoagulant drugs was reported in 89.2% of patients in the cardioversion group and 64.0% of patients in the non‐cardioversion group. Beta‐blocker use was reported in 59.6% of patients requiring cardioversion vs 54.3% of those not requiring cardioversion, and digoxin use in 23.6% vs 17.6%, respectively. Calcium channel blocker use was the same in both groups (~18%). The frequency of prior AAD use was high in both groups (∼70%) and without notable differences in AAD type. Rates of prior amiodarone use (cardioversion: 27.5%; non‐cardioversion: 30.8%), and discontinuation of prior AADs due to AEs (cardioversion: 15.1%; non‐cardioversion: 12.0%) and lack of efficacy (cardioversion: 33.2%; non‐cardioversion: 30.0%) were balanced between patients with and without baseline cardioversion. Within each cardioversion group, demographics and disease characteristics were generally similar for patients treated with dronedarone or placebo.

**Table 2:** Prior and concomitant medications

	Cardioversion			Non‐cardioversion		
Parameter	Dronedarone (n = 243)	Placebo (n = 121)	Total (n = 364)	Dronedarone (n = 585)	Placebo (n = 288)	Total (n = 873)
Study, n						
EURIDIS	153	75	228	258	126	384
ADONIS	90	46	136	327	162	489
Concomitant and prior medications						
Concomitant medication, n (%)^a^						
Beta‐blocker (except sotalol)	145 (59.7)	72 (59.5)	217 (59.6)	308 (52.6)	166 (57.6)	474 (54.3)
Digoxin	51 (21.0)	35 (28.9)	86 (23.6)	94 (16.1)	60 (20.8)	154 (17.6)
Calcium‐channel blocker (rate lowering)	41 (17.2)	24 (19.8)	65 (18.1)	98 (17.3)	54 (19.3)	152 (18.0)
Oral anticoagulant	215 (90.0)	106 (87.6)	321 (89.2)	356 (63.0)	185 (66.1)	541 (64.0)
Previous antiarrhythmic treatment, n (%)^b^, ^c^	165 (67.9)	87 (71.9)	252 (69.2)	440 (75.2)	211 (73.3)	651 (74.6)
Class IC	47 (19.3)	32 (26.4)	79 (21.7)	143 (24.4)	76 (26.4)	219 (25.1)
Amiodarone	59 (24.3)	41 (33.9)	100 (27.5)	184 (31.5)	85 (29.5)	269 (30.8)
Sotalol	67 (27.6)	37 (30.6)	104 (28.6)	147 (25.1)	75 (26.0)	222 (25.4)
Reasons for discontinuation of prior AADs^d^						
Adverse event	37 (15.2)	18 (14.9)	55 (15.1)	75 (12.8)	30 (10.4)	105 (12.0)
Lack of efficacy	71 (29.2)	50 (41.3)	121 (33.2)	171 (29.2)	91 (31.6)	262 (30.0)
Other medical reason	86 (35.4)	43 (35.5)	129 (35.4)	260 (44.4)	127 (44.1)	387 (44.3)
Patient request	8 (3.3)	10 (8.3)	18 (4.9)	45 (7.7)	23 (8.0)	68 (7.8)

Abbreviation: AAD, antiarrhythmic drug.

^a^ Data were missing for <4% of patients; percentages are calculated as a proportion of all patients with available data.

^b^ Patients could have taken more than one previous medication.

^c^ Other prior antiarrhythmic therapy included class IA, IB, II, and IV antiarrhythmic drugs.

^d^ Data were missing for <14% of patients.

### 3.2 Efficacy and clinical outcomes

**Figure 1:**
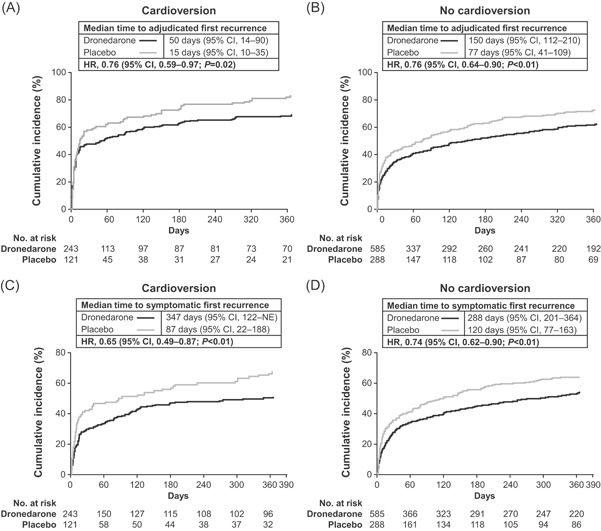
Kaplan–Meier unadjusted estimates of time to (A,B) adjudicated and (C,D) symptomatic first atrial fibrillation/flutter recurrence. CI, confidence interval; HR, hazard ratio, NE, not estimated

Treatment with dronedarone vs placebo was associated with reduced risk of adjudicated first AF/AFL recurrence by 24% regardless of baseline cardioversion status (Figure 1A,B). Median time to first AF/AFL recurrence was markedly shorter in the cardioversion group (50 days [95% CI, 14‐90] for dronedarone and 15 days [95% CI, 10‐35] for placebo; HR 0.76; 95% CI, 0.59‐0.97; *P* = .02) vs the non‐cardioversion group (150 days [95% CI, 112‐210] for dronedarone and 77 days [95% CI, 41‐109] for placebo; HR 0.76; 95% CI, 0.64‐0.90; *P* < .01). Dronedarone was associated with a lower ventricular rate vs placebo at the time of first AF/AFL recurrence regardless of symptoms (cardioversion: mean [SD], 93 bpm [26] vs 103 bpm [26] and median [range], 89 bpm [53‐217] vs 101 bpm [56‐168]; non‐cardioversion: mean [SD], 105 bpm [26] vs 115 bpm [32] and median [range], 103 bpm [46‐173] vs 109 bpm [55‐226]).

**Figure 2:**
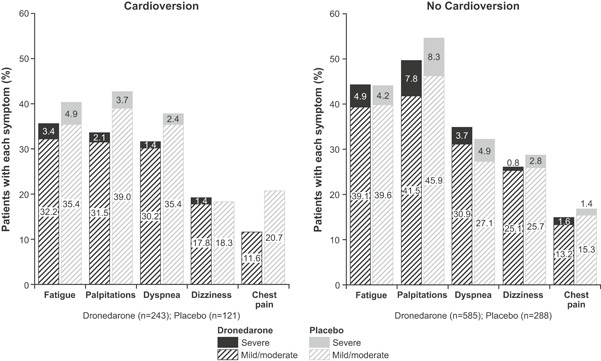
Frequency and severity of symptoms at first atrial fibrillation/flutter recurrence during 12 months of treatment

Median time to symptomatic first recurrence of AF/AFL was also longer with dronedarone vs placebo among patients requiring baseline cardioversion (347 vs 87 days, HR 0.65; 95% CI, 0.49‐0.87; *P* < .01) and those not requiring cardioversion (288 vs 120 days, HR 0.74; 95% CI, 0.62‐0.90; *P* < .01) (Figure 1C,D). A high proportion of documented first recurrences were symptomatic irrespective of cardioversion status, with a lower incidence in the dronedarone treatment arms of each group (cardioversion: dronedarone, 119/243 [49.0%]; placebo, 77/121 [63.6%]; non‐cardioversion: dronedarone, 302/585 [51.6%]; placebo, 178/288 [61.8%]). Fatigue and palpitations were the most common symptoms associated with AF/AFL recurrences regardless of cardioversion status, with the majority being of mild or moderate severity (Figure 2). Dronedarone was associated with a lower ventricular rate vs placebo at symptomatic first AF/AFL recurrence (cardioversion: mean [SD], 93 bpm [27] vs 107 bpm [27] and median [range], 88 bpm [53‐217] vs 107 bpm [58‐168]; non‐cardioversion: mean [SD], 107 bpm [28] vs 119 bpm [31], and median [range], 105 bpm [41‐208] vs 117 bpm [57‐226]).

**Figure 3:**
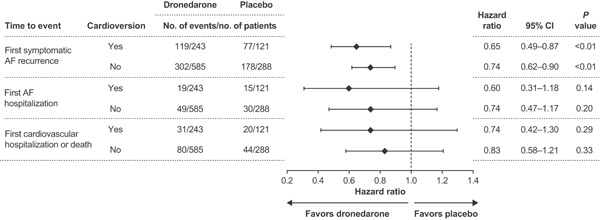
Risk of an event: pooled analyses of secondary endpoints in patients analyzed by cardioversion status. AF, atrial fibrillation; CI, confidence interval

In the cardioversion group, first cardiovascular hospitalization or death within 12 months occurred in 12.8% of patients receiving dronedarone vs 16.5% of patients receiving placebo (HR 0.74; 95% CI, 0.42‐1.30); among patients not requiring cardioversion, the corresponding frequencies were 13.7% vs 15.3% (HR 0.83; 95% CI, 0.58‐1.21). First AF hospitalization within 12 months occurred in 7.8% vs 12.4% of patients receiving dronedarone vs placebo, respectively, in the cardioversion group (HR 0.60; 95% CI, 0.31‐1.18) and in 8.4% vs 10.4% in the non‐cardioversion group (HR 0.74; 95% CI, 0.47‐1.17) (Figure 3).

### 3.3 Safety

In the primary analysis of the EURIDIS and ADONIS trials, hepatobiliary events were infrequent (<2%) and similar across treatment groups. Elevations in liver enzymes were similar among patients treated with dronedarone (12.2%) vs placebo (13.6%).^8^ Small elevations of serum creatinine (approximately 10 µmol/L) were reported in 2.4% of patients treated with dronedarone vs 0.2% treated with placebo.^8^ Incidences of TEAEs, serious TEAEs, TEAEs leading to treatment discontinuation, and deaths in the current analysis by cardioversion status are summarized in Table 3. Deaths due to any cause from first study drug intake up to 10 days after last study drug intake were reported in eight patients treated with dronedarone (two cardioverted and six non‐cardioverted) and three patients treated with placebo (all non‐cardioverted). Prior or concomitant digoxin use was reported in five of the 11 patients who died: four in patients treated with dronedarone (one cardioverted and three non‐cardioverted) and one in a patient treated with placebo (non‐cardioverted). Five of the patients who died also had a history of structural heart disease; four of these were treated with dronedarone (all non‐cardioverted) and one with placebo (non‐cardioverted).

**Table 3:** Summary of TEAEs and incidence of TEAEs reported in ≥3% of patients in any group

	Cardioversion		Non‐cardioversion	
TEAEs (n)(%)	Dronedarone (n = 243)	Placebo (n = 121)	Dronedarone (n = 585)	Placebo (n = 288)
Summary of TEAEs				
Any TEAE	156 (64.2)	80 (66.1)	422 (72.1)	189 (65.6)
Any serious TEAE	47 (19.3)	31 (25.6)	117 (20.0)	69 (24.0)
Death (any cause)	2 (0.8)	0	6 (1.0)	3 (1.0)
Sudden death	0	0	4 (0.7)	1 (0.3)
Any TEAE leading to study discontinuation	21 (8.6)	7 (5.8)	59 (10.1)	22 (7.6)
TEAEs with incidence ≥3%				
Cardiac disorders				
Atrial fibrillation	16 (6.6)	14 (11.6)	45 (7.7)	27 (9.4)
Bradycardia	10 (4.1)	5 (4.1)	11 (1.9)	2 (0.7)
Angina pectoris	5 (2.1)	4 (3.3)	14 (2.4)	6 (2.1)
Gastrointestinal disorders				
Diarrhea	16 (6.6)	5 (4.1)	47 (8.0)	17 (5.9)
Nausea	7 (2.9)	2 (1.7)	29 (5.0)	12 (4.2)
General disorders and administration site conditions				
Peripheral edema	13 (5.3)	7 (5.8)	26 (4.4)	13 (4.5)
Fatigue	5 (2.1)	2 (1.7)	16 (2.7)	9 (3.1)
Respiratory, thoracic, and mediastinal disorders				
Dyspnea	8 (3.3)	5 (4.1)	14 (2.4)	8 (2.8)
Cough	8 (3.3)	3 (2.5)	11 (1.9)	4 (1.4)
Nervous system disorders				
Headache	11 (4.5)	9 (7.4)	33 (5.6)	21 (7.3)
Dizziness	3 (1.2)	5 (4.1)	20 (3.4)	3 (1.0)
Musculoskeletal and connective tissue disorders				
Back pain	6 (2.5)	1 (0.8)	23 (3.9)	7 (2.4)
Arthralgia	3 (1.2)	3 (2.5)	19 (3.2)	3 (1.0)
Muscle spasms	3 (1.2)	4 (3.3)	9 (1.5)	3 (1.0)
Infections and infestations				
Nasopharyngitis	5 (2.1)	4 (3.3)	17 (2.9)	5 (1.7)
Upper respiratory tract infection	5 (2.1)	1 (0.8)	24 (4.1)	4 (1.4)
Vascular disorders				
Hypertension	4 (1.6)	1 (0.8)	15 (2.6)	9 (3.1)

Abbreviation: TEAE, treatment‐emergent adverse event.

## 4 DISCUSSION

In this analysis of the EURIDIS and ADONIS studies, we evaluated patient characteristics, patterns of AF/AFL recurrences, clinical outcomes, and safety in patients with nonpermanent AF/AFL by baseline cardioversion status. We observed a number of important differences in patients requiring cardioversion vs those who did not. Patients requiring cardioversion had a greater prevalence of underlying cardiovascular disease, structural heart disease, and congestive heart failure, had larger left atrial diameter, and were more likely to have a CHA_2_DS_2_‐VASc score of ≥2; this was consistent with the greater use of oral anticoagulant drugs in these patients compared with those in the non‐cardioversion group. While both groups had similar sinus rates at baseline, patients requiring cardioversion had lower ventricular rates at last AF/AFL before randomization and at first AF/AFL recurrence, which may be due to the overall more frequent use of digoxin and beta‐blockers in this group.

Despite differences in comorbidities and background medications, the relative efficacy of dronedarone compared with placebo in reducing first AF/AFL recurrence was similar regardless of baseline cardioversion status (*P* value for interaction = .87).^8^ Similarly, dronedarone was associated with prolonged time to symptomatic first AF/AFL recurrence relative to placebo irrespective of cardioversion status, and was associated with a trend toward lower incidence of first cardiovascular hospitalization or death within 12 months as well as AF hospitalization within 12 months. The mean ventricular rates at first adjudicated and symptomatic AF/AFL recurrences in patients treated with dronedarone vs placebo were lower by 10 to 14 bpm across cardioversion groups.

Patients with baseline cardioversion had markedly shorter times to first AF/AFL recurrence compared with patients without cardioversion (dronedarone, 50 vs 150 days; placebo, 15 vs 77 days). A possible contributor to this observation is that patients requiring baseline cardioversion appear to have had more advanced cardiac abnormalities including a higher prevalence of heart failure, structural heart disease, left atrial enlargement, and potentially, persistent AF pre‐cardioversion.^10^, ^11^, ^12^

Time to symptomatic AF/AFL was longer in the cardioversion group than in the non‐cardioversion group among patients treated with dronedarone (347 vs 288 days) but not placebo (87 vs 120 days). The frequency of symptoms at recurrence was lower among patients treated with dronedarone than placebo in both cardioversion groups. The rate‐lowering properties of dronedarone in conjunction with the greater use of beta‐blockers and digoxin in patients requiring cardioversion may explain these findings, which are also in line with reports that symptomatic AF is more likely to occur in patients with higher ventricular rates.^13^ In addition, this observation suggests that the extent of AF‐associated symptoms may not be directly associated with overall AF burden (and type of AF, ie, paroxysmal vs persistent). Although perhaps seemingly paradoxical, similar observations have been reported in previous AF studies.^14^, ^15^, ^16^

The present analysis identified no new safety concerns with regard to dronedarone treatment in patients requiring cardioversion before AAD treatment and demonstrated that the safety of dronedarone was comparable in patients with or without cardioversion. Few deaths were observed, and there was no imbalance between the cardioversion and non‐cardioversion groups. In addition, taking into account the 2:1 randomization of dronedarone and placebo, respectively, the rate of deaths was comparable in both treatment arms.^8^ Safety data from the primary EURIDIS/ADONIS analysis show that hepatobiliary events and elevations in liver enzymes were similar among patients treated with dronedarone vs placebo; our observations with regard to liver safety are aligned with recent real‐world evidence.^17^ Elevations of serum creatinine were consistent with prior reports, and are attributed to the inhibition of tubular secretion of creatinine and not to glomerular filtration rates.^7^, ^18^

Prior AF studies evaluating AADs neither characterized nor were designed to address potential variable effects of AADs after cardioversion. In the Canadian Trial of Atrial Fibrillation^1^ (CTAF; amiodarone vs sotalol or propafenone) and the Sotalol Amiodarone Atrial Fibrillation Efficacy Trial^2^ (SAFE‐T; amiodarone vs sotalol) in patients in either sinus rhythm or with AF at randomization, cardioversion was performed in patients with AF who did not convert to sinus rhythm after randomization and AAD initiation. No data on disposition or characteristics based on cardioversion status have been reported for these studies. In the Rythmol Atrial Fibrillation Trial (RAFT), which evaluated variable doses of sustained‐release propafenone vs placebo, patients were required to be in sinus rhythm before initiation of AAD therapy, with electrical cardioversion performed as needed.^5^ While the efficacy of propafenone was lower in patients with vs without a history of cardioversion, the sample size of patients with cardioversion was small (approximately 20‐30 patients per dose group), and patient characteristics, the timing of cardioversion, and overall safety experience based on cardioversion status were not reported. The EURIDIS and ADONIS studies, with a combined size of >1200 patients, the extent of baseline cardioversion (~30% of patients), and inclusion of cardioversion as a randomization variable, are uniquely designed to better assess this clinical question.^8^

A few important caveats and limitations to our analysis are to be noted. Due to the post hoc nature of our analysis, our results should be considered exploratory. Our observations with dronedarone may not reflect findings with other AADs. While cardioversion status was subject to stratification in the EURIDIS and ADONIS studies to ensure balance within treatment arms, the studies were not powered to evaluate efficacy and clinical outcomes based on cardioversion status. Multivariate analyses to identify factors associated with AF/AFL recurrence could not be performed, and data on more remote cardioversion history, AF/AFL burden, and asymptomatic AF/AFL recurrence were not available. Finally, it is possible that an imbalance with regard to the type of AF/AFL (ie, higher proportion of patients with persistent AF/AFL among patients requiring cardioversion) was operative in the study population. However, AF type was not characterized in the EURIDIS and ADONIS studies. This limitation may reflect the time when these studies were designed and initiated in 2001. In line with this, the 2001 ACC/AHA/ESC AF guidelines, the first of such guidelines, introduced specific definitions of AF types.^19^ In 2003, the North American Society of Pacing and Electrophysiology, the precursor to the Heart Rhythm Society, published a consensus paper on AF nomenclature and classification describing AF types.^20^ Definitions of AF types continue to evolve today.^21^, ^22^, ^23^, ^24^

## 5 CONCLUSIONS

Our analysis with dronedarone sought to better understand the characteristics and outcomes of patients with nonpermanent AF/AFL requiring cardioversion who are candidates for AAD therapy. Important differences were observed between patients who required cardioversion vs those who did not; these included a greater prevalence of underlying cardiovascular disease and structural heart disease among patients requiring cardioversion. In addition, patients requiring cardioversion experienced substantially shorter times to first AF/AFL recurrence compared with patients not requiring cardioversion. Taken together, these results underscore that patients requiring cardioversion for management of AF/AFL constitute a higher‐risk population; understanding the benefits and risks of AAD treatment in this patient population is of considerable clinical interest. Dronedarone delayed adjudicated and symptomatic AF/AFL recurrence regardless of baseline cardioversion status. The safety profile of dronedarone was also similar in patients with and without baseline cardioversion despite baseline differences in comorbidities. Future prospective studies of dronedarone, as well as other AADs, are warranted to determine the efficacy and safety of AAD therapy post‐cardioversion.
